# Optimising Translational Research Opportunities: A Systematic Review and Narrative Synthesis of Basic and Clinician Scientists' Perspectives of Factors Which Enable or Hinder Translational Research

**DOI:** 10.1371/journal.pone.0160475

**Published:** 2016-08-04

**Authors:** Nina Fudge, Euan Sadler, Helen R. Fisher, John Maher, Charles D. A. Wolfe, Christopher McKevitt

**Affiliations:** 1 Division of Health and Social Care Research, Faculty of Life Science and Medicine, King’s College London, London, United Kingdom; 2 National Institute for Health Research (NIHR) Comprehensive Biomedical Research Centre, Guy's and St Thomas' NHS Foundation Trust and King's College London, London, United Kingdom; 3 Centre for Implementation Science, Department of Health Service and Population Research, Institute of Psychiatry, Psychology & Neuroscience, King’s College London, London, United Kingdom; 4 Department of Research Oncology, King’s College London and Guy’s Hospital, London, United Kingdom; 5 Department of Clinical Immunology and Allergy, King’s College Hospital, London, United Kingdom; University of British Columbia, CANADA

## Abstract

**Introduction:**

Translational research is central to international health policy, research and funding initiatives. Despite increasing use of the term, the translation of basic science discoveries into clinical practice is not straightforward. This systematic search and narrative synthesis aimed to examine factors enabling or hindering translational research from the perspective of basic and clinician scientists, a key stakeholder group in translational research, and to draw policy-relevant implications for organisations seeking to optimise translational research opportunities.

**Methods and Results:**

We searched SCOPUS and Web of Science from inception until April 2015 for papers reporting scientists’ views of the factors they perceive as enabling or hindering the conduct of translational research. We screened 8,295 papers from electronic database searches and 20 papers from hand searches and citation tracking, identifying 26 studies of qualitative, quantitative or mixed method designs. We used a narrative synthesis approach and identified the following themes: 1) differing concepts of translational research 2) research processes as a barrier to translational research; 3) perceived cultural divide between research and clinical care; 4) interdisciplinary collaboration as enabling translation research, but dependent on the quality of prior and current social relationships; 5) translational research as entrepreneurial science. Across all five themes, factors enabling or hindering translational research were largely shaped by wider social, organisational, and structural factors.

**Conclusion:**

To optimise translational research, policy could consider refining translational research models to better reflect scientists’ experiences, fostering greater collaboration and buy in from all types of scientists. Organisations could foster cultural change, ensuring that organisational practices and systems keep pace with the change in knowledge production brought about by the translational research agenda.

## Introduction

The term translational research has been in use for over 30 years, but has really come into focus in the health field in the last ten years and is now central to international health policy, research and funding initiatives [1]. Translational research has been characterised as harnessing the use of discoveries from basic science to develop new diagnostic tests, therapies and prevention devices (sometimes referred to as T1 type translation), as well as the implementation of research findings into practice to improve care for patients (T2 type translation) [2]. The need for translational research is based on the premise that much research in the life sciences has failed to advance human health, and it offers itself up as a solution to tackle intractable health problems [3].

Although accorded much prominence internationally, the translation of basic science findings into clinical practice is not straightforward. A substantial number of editorials, opinion pieces and policy documents make reference to barriers to translational research. These barriers include: a lack of a ‘culture of translation’ within institutions [4, 5]; inadequate infrastructure, including a lack of facilities to conduct clinical research [2, 5]; and an inadequately trained workforce and difficulties retaining those who do possess the necessary skills [4, 6, 7]. Collaboration is proposed as a key requirement for translational research with suggestions that it is inhibited by the compartmentalisation of departments within universities and hospitals, a cultural divide between scientists and clinicians, and a university system that rewards individual achievement rather than joint working practices [4–6, 8]. At the policy level, a number of initiatives have been established with the aim to reduce perceived bottle necks in translational research in order to accelerate the translation of scientific knowledge into effective health measures with health benefits for patients and wealth benefits for the nation [9–12]. For example, in the US, Clinical and Translational Science Awards (CTSA) fund the development of innovative solutions to improve the efficiency, quality and impact of the translational research processes. In England, the creation of biomedical research centres brings together those working in a hospital setting with those in a university setting. However, less is known about the challenges and enablers of translational research from the perspective of those largely held responsible for conducting translational research: basic and clinician scientists. A growing body of empirical research has begun to address this gap.

To date, translational research has been positioned as bridging two seemingly disparate worlds: basic science and clinical medicine, with the former assumed to inform and feed into the latter. However, a number of commentators have challenged this view, affirming the huge diversity of activities within translational research, and pointing out that, although often framed as a singular ‘bench to bedside’ concept, translational research actually consists of multiple forms and processes which vary by discipline, institution and country [3, 13–15]. This also underscores the importance of studies informed by the social sciences which until recently have paid only limited attention to translational research [13]. Scholars have begun to theorise how translational research is defined, what the organisation of health research into translational research models can tell us about biomedicine today, and how institutional practices shape visions for translational research [3, 16]. These authors point to a number of factors shaping translational research, including the increased bureaucratisation of the university, the influence of an audit culture on structuring research, and the increased capitalisation of the life sciences.

The review team consisted of social scientists and clinician scientists, affiliated to a translational research organisation, charged with understanding methods to improve translational research processes. We argue that synthesising the growing body of empirical research on the views of basic and clinician scientists can shed light on how to optimise current policy agendas of translational research, but also provides empirical evidence to address broader questions about translational research as a concept. The aim of our investigation is first, to systematically review and synthesise studies examining factors enabling or hindering translational research from the perspective of basic and clinician scientists; and second, to use these findings to inform policy at the institutional level to better realise the potential for translational research.

## Methods

### Synthesis approach

We conducted a narrative synthesis of available papers that examined scientists’ perspectives of the factors which enable or hinder translational research. Narrative synthesis is an established method providing a rigorous framework for systematically reviewing and synthesising emerging conceptual themes from studies, which can be of qualitative, quantitative or mixed designs [17]. The approach aims to produce a textual, narrative understanding of findings from included studies conducted in different settings and contexts. It is suited to a field of enquiry where little is known, and aims to synthesise findings from studies in order to generate new knowledge, and critique existing concepts. This approach was considered particularly useful to examine themes related to factors perceived to inhibit or enable translational research from the perspective of scientists.

### Selection criteria

We included primary research studies published in peer reviewed English language journals that used qualitative, quantitative or mixed methods to examine factors which hinder or enable translational research, from the perspective of clinician and basic scientists. We excluded editorial sources as these are essentially opinion or commentary from one individual. Our intention was to review the growing empirical research in this field which not only reports views of scientists but subjects them to conceptually and contextually based critical analysis. For the purposes of this review, our definition of translational research focused on research at the interface of laboratory and clinical research, although authors and their research participants did not have to explicitly use the term ‘translational research’. Studies were excluded if they: defined translational research as the implementation of research findings into practice as this area is conceptually different from T1 type translational research [2] and has been the focus of numerous reviews and meta-reviews to date [18–22]; did not discuss factors enabling or hindering translational research; only considered patients’ perspectives of translational research; or solely explored difficulties with recruiting participants to trials, as this topic has been the focus of several reviews already [23, 24].

#### Search strategy

**Fig 1:**
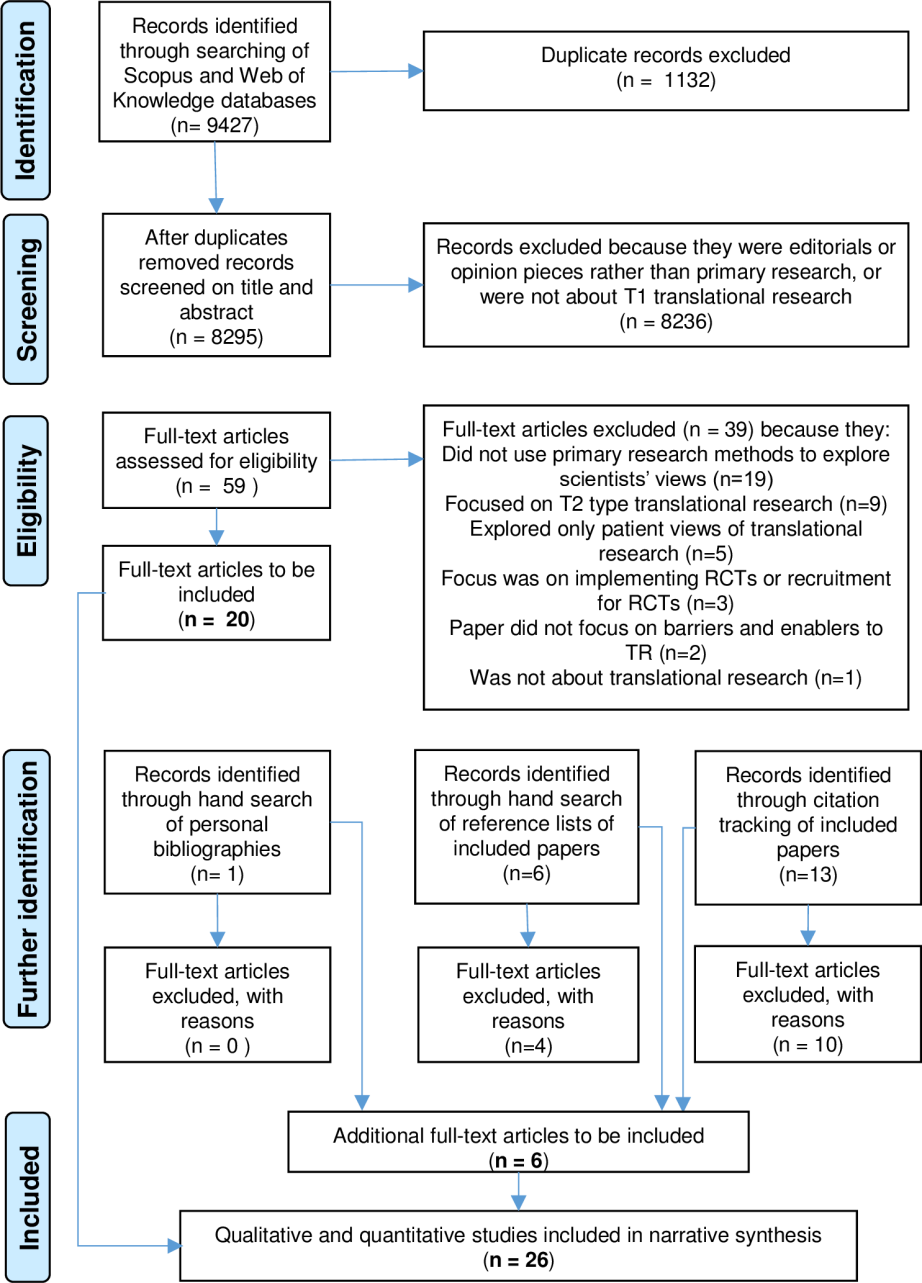
Flow diagram of the different phases of the systematic search and review based on PRISMA [26].

The search strategy utilised a range of systematic and serendipitous methods to identify relevant studies [25]. First, we searched two electronic database platforms–SCOPUS and Web of Knowledge–from inception until April 2015. These were searched in the title, abstract and keywords using key terms for translational research combined with thesaurus and free text terms for the subject areas of health and medicine (see Table 1). The titles and abstracts of returned articles were scrutinised and full text articles obtained for papers likely to meet the inclusion criteria. Each potentially relevant article was retrieved and read in full by two authors to determine whether it met the inclusion criteria. After identifying relevant papers through database searching, the reference lists of retrieved articles were searched for other studies which might meet the inclusion criteria. We searched personal biographies of experts in the field. Finally, we undertook citation tracking in Google Scholar of all included papers, to identify any additional studies. Fig 1 illustrates the flow of studies through the stages according to PRISMA [26].

**Table 1:** Search strategy.

Terms for translational research searched in title, abstract and key words	“bench to bedside” OR “translational research” OR “knowledge production” OR “knowledge transfer” OR “knowledge broker”
Terms for health and medicine searched in searched in title, abstract and key words	health* OR medicine

#### Quality appraisal

Two authors (NF, ES) independently appraised the quality of each study using a five-point checklist comparing quality scores given to each paper to reach a consensus [27]. This checklist assesses the methodological quality of studies and can be applied to empirical papers regardless of study design. Quality appraisal involved scoring each paper out of five according to how well the following criteria were met:

1. Are the aims and objectives of the research clearly stated?
2. Is the research design clearly specified and appropriate for the aims and objectives of the research?
3. Do the researchers provide a clear account of the process by which their findings were produced?
4. Do the researchers display enough data to support their interpretations and conclusions?
5. Is the method of analysis appropriate and adequately explicated?

Papers scoring four or five were considered to be of high quality. Papers scoring 1 to 3 only partially met the five criteria and were judged to be of low quality. Given the lack of consensus surrounding the use of quality appraisal in qualitative research, with suggestions that appraisal scores can be more reflective of the written report rather than the actual study and that there is a risk of discounting important studies for the sake of ‘surface mistakes’ [28, 29], we did not exclude studies with low quality scores. Instead we used this quality appraisal to assess the robustness of the synthesis.

### Data extraction and method of synthesis

Following the guidance developed by Popay et al., we first used tabulation and thematic analysis to systematically extract and synthesise data from included studies [17]. NF and ES constructed tables, adding relevant information from each included paper under the following column headings: country, field of translational research, methods, definition and model of translational research, theoretical approach and main findings. Once tabulation was complete NF and ES used thematic analysis, guided by the principles of the constant comparative method [30], to inductively look for similarities and differences across the studies, grouping these patterns and relationships into conceptual themes. We used the ‘one sheet of paper method’ to visually map out themes and subthemes [31]. Themes and subthemes identified through the synthesis were then reviewed by the remaining authors in order to check for consistency in the data brought together under a theme.

## Results

Our search generated a total of 9427 articles, of which 1132 duplicates were removed. The papers were then screened based on title and abstract, with 8236 papers excluded for not meeting the inclusion criteria. Studies were mainly excluded because they did not focus on T1 type translational research or because they discussed translational research from an editorial or opinion piece perspective rather than being based on empirical research. Full text articles of 59 studies were retrieved and read in full. Thirty-nine papers were excluded primarily because they were not empirical studies, with other reasons for exclusion listed in Fig 1. Six additional papers were identified through: hand searching reference lists of included studies (n = 2), personal biographies of experts in the field (n = 1), and citation tracking of included studies (n = 3) (see Fig 1).

### Overview of included studies

The 26 papers included in the synthesis focused on studies investigating enablers and barriers to translational research from the perspectives of basic and clinician scientists undertaking translational research across nine countries (see Table 2 for a summary of the included papers). The majority of papers reported on translational research from the USA (n = 10), with papers also from Canada (n = 4), UK (n = 4), Australia (n = 1) and China (n = 3). In addition, four papers compared translational research across a number of countries: Austria, Finland and Germany; Germany and USA; UK and USA; UK and Germany. The dominance of western countries covered by studies included in our review is perhaps not surprising given our inclusion requirement that papers were published in the English language and the importance of translational research in the Anglo-American biomedical audit and funding cultures [3]. The earliest paper was published in 1998 and the most recent in 2014, with the majority (n = 21) being published in the six years between 2009 and 2014. This is indicative of the increased interest in translational research and policy concerns with improving the process of translating biomedical knowledge and innovation into clinical benefit [13, 14]. The majority of papers (n = 17) were concerned with translational research undertaken in university or hospital settings or as part of institutions which combined university and clinical facilities, such as academic health science centres, translational research organisations or research networks. The papers used a range of quantitative and qualitative methods to investigate scientists’ views of translational research, with six using a survey and eight using semi-structured interviews. Other methodological approaches were case studies (n = 6), ethnographic (n = 4) and documentary analysis (n = 2). Of the 20 papers which adopted a qualitative approach to data collection and analysis, 14 studies explicitly incorporated a theoretical approach through which to collect, analyse and interpret the data. Incorporating a theoretical perspective enhances the quality of qualitative research; allowing understanding and contextualisation of translational research as a dynamic, negotiated, and situated entity or construct. Authors of these 14 studies either situated their approach in a sub-field of a social science discipline or specifically indicated their theoretical perspective. The following theories and concepts were drawn on: symbolic interactionism (n = 1), argumentative policy analysis (n = 1), Bourdieu’s logic of practice (n = 1), Gieyrn’s boundary work and the concept of therapeutic misconception (n = 1), Wittgenstein’s rules for the interpretation of a rule (n = 1), the sociology of professions (n = 1), the sociology of expectations (n = 2), entrepreneurial science (n = 2), and theoretical concepts on innovation (n = 4). Two studies using quantitative survey methods also located their data within theoretical frameworks related to the concept of team science and theoretical concepts from the management field.

**Table 2:** Characteristics and summary of studies included in the review.

Reference	Country	Health domain	Setting	Method and participants	Theoretical approach	Quality appraisal score
Campbell et al. (2001) [32]	USA	Not specified	Hospital-University collaboration	Quantitative survey of 478 (Response Rate (RR) 67.1%) department chairs and senior research administrators in US medical schools to assess quality of clinical research and its challenges.	─	5
Chen (2009) [33]	China	Stem cells	Hospital-University collaboration, biotech company	Interviews (n = 11) to obtain overview of stem cell research in China, followed up by case studies of three sites of stem cell research entailing interviews, observation of meetings, analysis of documents.	─	2
Etzkowitz (1998) [34]	USA	Translational research (focusing on biology, computer science, electrical engineering, physics and chemistry)	University	Longitudinal case studies including in-depth interviews of two universities newly involved with industry to investigate the effects of new university/industry linkages on the way scientists view research, interpret the scientific role and interact with colleagues, companies and universities.	Entrepreneurial science	2
French & Miller (2012) [35]	Canada	Not specified	Hospital-University collaboration	Semi-structured interviews with 26 key informants working within an academic health science system to explore healthcare oriented and healthcare based innovation.	Entrepreneurial science	5
Hallowell et al. (2009) [36]	UK	Cancer genetics	Clinics (not specified)	Interviews with 40 healthcare professionals or academic researchers involved in research to investigate relationships within a research programme.	Boundary work and therapeutic misconception	4.5
Harris et al. (2012) [37]	USA	Cancer	Research Network	Survey of 18 (RR = 85.7%) representatives from organisations involved in a cancer research network across Arizona state to establish benefits and drawbacks of collaboration.	Team science and social network theory	4
Heller et al. (2009) [38]	USA	Not specified	Hospital-University collaboration	Documentary analysis of 12 NIH Clinical and Translational Science Awards written by scientists applying for institutional level initiatives to speed up translational research processes. The analysis investigated to what extent solutions proposed in the awards addressed barriers to translational research as identified in the literature.	─	4.5
Kahn et al. (2011) [39]	USA	Not specified	Clinics (not specified)	Interviews with 243 clinicians (physicians, dentists, nurse practitioners) and other key stakeholders to examine feasibility of research participation in their own community clinical settings.	─	4
Kotarba et al. (2013) [40]	USA	Not specified	Hospital-University collaboration	Interviews with 39 scientists, clinicians and administrators involved with NIH CTSA research projects to examine the everyday reality of translational science research.	Symbolic interaction	3
Lander & Atkinson-Grosjean (2010) [41]	Canada	Pathogenomics of innate immunity	Hospital-University collaboration	Case-study research methods (surveys, semi-structured interviews (n = 20)) to understand the barriers for career entry and progress perceived by clinician-scientists and to explore whether these perceived barriers are supported in Canadian Institutes of Health Research data on grant and award performance of clinician scientists and non-clinical scientists.	Innovation systems	3.5
Lander et al. (2011) [42]	Canada	IRAK-4 deficiency	Hospital-University collaboration	A nested case study of two laboratories run by clinician-scientists to identify translational practices mediating clinical and research goals of the laboratory team. Data collection entailed structured survey interviews (n = 20) with follow up semi-structured interviews; and participant observation of day-to-day laboratory operations.	Research and innovation	3.5
Long et al. (2014) [43]	Australia	Cancer	Research network	Survey of 52 (RR = 76.4%) hospital-based clinicians and university-based researchers to examine patterns of collaboration.	Collaboration theories from management literature: homophily, proximity, trust, reputation	5
Morgan et al. (2011) [44]	UK	Not specified	Hospital-University collaboration	Informed by an ethnographic approach, data collection entailed semi-structured interviews (n = 24) and exploratory interviews with a ‘research translator’ and clinical and basic scientists, documentary evidence and observation of research meetings.	Bourdieu’s logic of practice	5
Ostergren et al. (2014) [45]	USA	Addiction	Research network	Semi-structured interviews with 20 scientists working in the field of genetics and addiction to explore their perspectives on the challenges and pressures of translating research findings into clinical practice and public health policy.	─	5
Payne et al. (2005) [46]	USA	Informatics as applied to translational research—health domains not specified.	Hospital-University collaboration	27 semi-structured interviews with clinical researchers and IT specialists involved in research to understand interaction patterns between clinician scientists and informaticians and how IT-based solutions are applied in translational research	─	2
Payne et al. (2013) [47]	USA	Informatics as applied to translational research—health domains not specified.	Hospital-University collaboration	Structured survey of 31 experts bioinformatics, computer science, information technology at Academic Health Centres (AHC) followed by thematic analysis of public-domain documents provided by AHCs.	─	4
Salazar et al. (2011) [48]	USA	Translational research across a range of health domains: allergy and immunology, cancer, genomic medicine.	Hospital-University collaboration	Online survey of 233 (RR = 28%) medical centre faculty about their participation in a disease-based interdisciplinary research team.	─	4
Stephens et al. (2013) [49]	UK	Stem cells	Laboratories (not specified)	Ethnographic case studies of laboratories dealing with human cellular material to investigate the tensions scientists face to establish particular levels of laboratory sterility suitable for handling cell therapies for clinical use.	Wittgenstein rules of interpretation;Collins ‘experimental method’; Pinch & Bijker ‘technological development’	4.5
Vignola-Gagné et al. (2013) [50]	Austria, Finland, Germany	Molecular medicine and genomics	Not specified	Documentary analysis of initiatives and policies dealing with translational research (policy formulations, government white papers, 200 editorials and reviews in peer reviewed journals) and 26 semi-directed interviews with policy makers and biomedical researchers to examine current translational practices and initiatives in the three countries.	Research innovation concepts	2
Vignola-Gagné (2013) [51]	Germany, USA	Not specified	Not specified	Case study of clinician-scientists in Germany and USA (documentary evidence, 35 semi structured interviews) to investigate the formulation and implementation of translational research as an emerging biomedical policy priority.	Argumentative policy analysis	4
Wainwright et al. (2006) [52]	UK	Stem cells for diabetes therapy	Laboratories (not specified)	Observation and informal interaction with scientists in a beta cell laboratory, interviews with seven of the 15 scientists working in the laboratory to explore their views on the prospects and problems of translational research in the field of stem cell science.	Sociology of expectations	4.5
Wainwright et al. (2008) [53]	UK, USA	Stem cells within neuroscience and diabetes	Laboratories and clinics (not specified)	In-depth interviews with 60 scientists and clinicians in leading stem cell labs and clinics in UK and USA exploring their views on the bench-bedside interface.	Sociology of expectations	5
Weston, et al. (2010) [54]	USA	Not specified	Hospital-University collaboration	Survey of 1800 (RR = 47%) faculty and postdoctoral fellows at John Hopkins Schools of Medicine, Public Health, Nursing and Engineering to investigate barriers to translational research.	─	4
Wilson‐Kovacs & Hauskeller.(2012) [55]	UK, Germany	Stem cells for cardiac repair	Laboratories (not specified)	Ethnographic approach entailing fieldwork in clinics undertaking clinical trials with autologous stem cells for cardiac repair, observations at scientific meetings, in-depth semi-structured interviews with clinician-scientists to explore how participants’ portrayed, explained and justified their role within the wider clinical research environment.	Sociology of professions	5
Zhang (2011)[56]	China	Stem cells	Scientific institutions	Interviews with 48 key stakeholders active in stem cell research to examine how the structure of scientific institutions affects effective governance.	Innovation	3
Zhou et al. (2013) [57]	China	Not specified	Hospital-University collaboration	Qualitative, multiple case study approach (including interviews and review of secondary sources) to assess the challenges faced by Translational Research Organisations.	─	2

**Fig 2:**
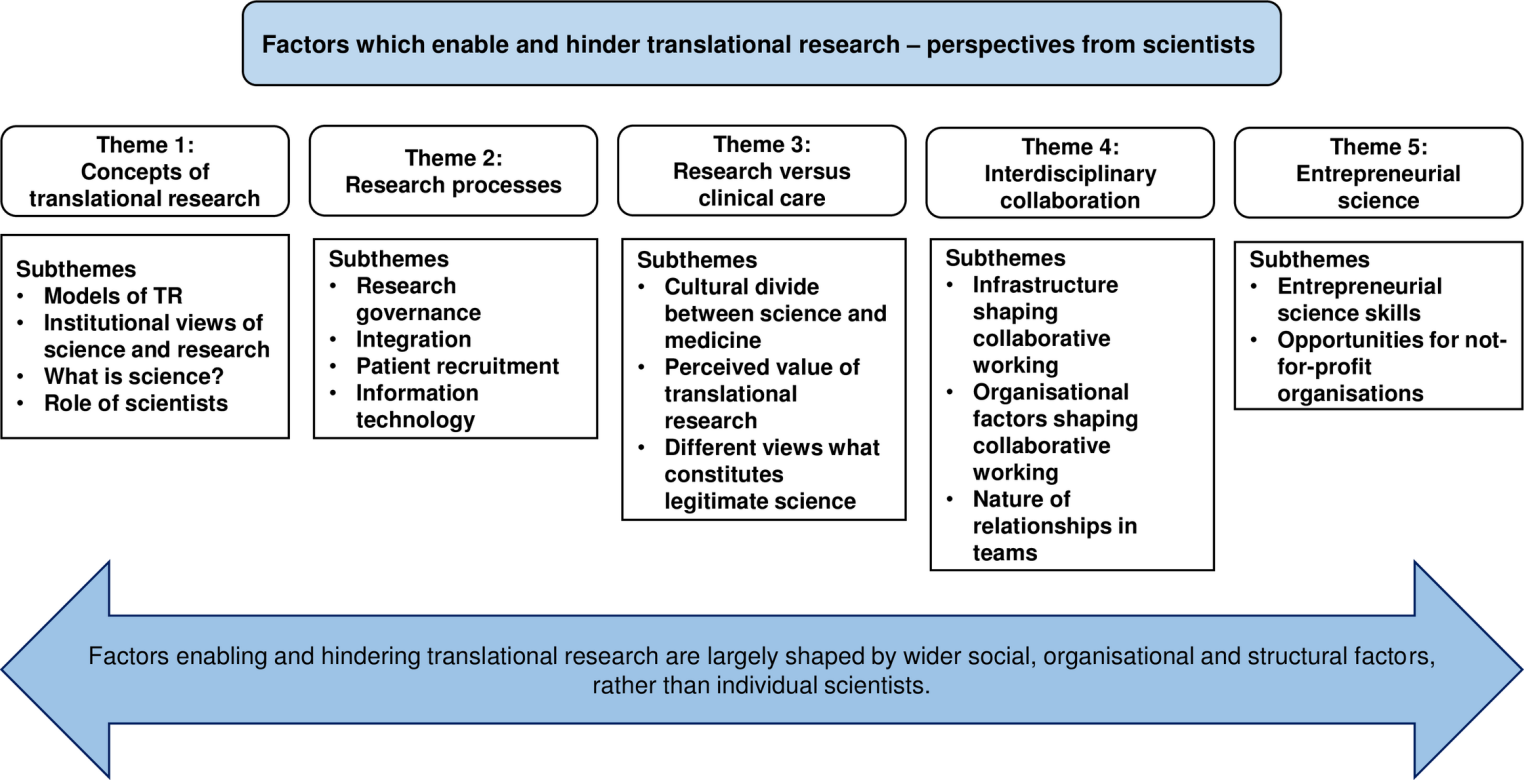
Themes and subthemes identified from the reviewed literature.

We identified five main conceptual themes representing factors which enabled or hindered the practice of translational research. The thematic categories and how they interrelate are discussed in detail in the sections below and are visually summarised in Fig 2. Within each thematic category illustrative supporting quotations are provided.

#### Theme 1: Concepts of translational research

Only one study in our sample explicitly looked at how scientists defined and understood the concept of translational research [44]. Morgan et al. conducted interviews with basic and clinician scientists in 2008 at a time when the ‘*requirements of translational research were only beginning to emerge*’ [44, p.948]. Both types of scientists reported awareness of the concept and its current policy emphasis, describing it as the ‘*mantra of the moment*’, but were unclear as to its meaning, and were able to give only a minimal definition such as ‘*to try to move stuff from the lab to the clinic*’ [44, p.948]. The other included studies, particularly those which utilised survey methods for data collection, did not report explicitly asking respondents to define translational research.

The type of translational research model adopted by an organisation or institution was perceived by scientists in a number of studies in our sample to influence the practice of translational research, with a linear model seen as an impediment to successful practice [33, 34, 40, 45, 53]. Institutional representation of translational research as a ‘pipeline’, and a process which requires acceleration, was articulated by basic and clinician scientists as problematic for a number of reasons: that science was seen as being reduced to solving problems and producing cures rather than discovering new knowledge [45, 53]; a lack of recognition that good science takes time [40, 45, 53]; and rapid selling of biomedical innovations to the public due to pressures to translate findings quickly for patient benefit without thinking through the implications of these developments or making better use of existing policies and interventions [34, 40, 45, 53]. For example, scientists working in the field of addiction research acknowledged that: ‘*translation takes time*, *that bodies of knowledge are built slowly over many years*, *and that basic science has value even in the absence of swift translation*’ [45, p.4].

These views contrast with institutional and policy interpretations of translational research which assume a linear model, impeded by ‘blocks’ that act as barriers to translating laboratory discoveries into improvements in human health [for example see 7]. The methods adopted by researchers in our sample allowed scientists to present a more subtle and nuanced view of the problems attributed to translational research and how they should be addressed [40, 51]. For example, basic and clinician scientists interviewed in one US study described challenges of translational research as ‘dilemmas’ as opposed to the oversimplified notion of ‘barriers’ and ‘blocks’ typically perceived by external agencies, including policy makers and research funders [40]. The preferred term ‘dilemmas’ indicates that there are sometimes very valid and important reasons why things do not unfold according to the expectations that policy makers and research funders may have.

Viewing translational research as a circular or iterative process, was seen to facilitate translational research practices by encouraging reciprocal interactions between the lab and the clinic. This was thought to promote a collaborative research environment, which in turn attracted basic and clinician scientists who wanted to collaborate (this is elaborated upon further in the theme ‘interdisciplinary collaboration’) [34, 41, 52, 57]. A case study of eight translational research organisations (TRO) in China elaborates on this point. In this study, TROs were established primarily by biomedical organisations. This limited the potential for TROs to address intractable problems because key disciplines required for successful translation, such as public health, health policy, social sciences, community engagement, had not been included [57].

#### Theme 2: Research processes

Research processes were perceived by basic and clinician scientists across a number of studies to enable or hinder translational research practices. Research processes included: regulatory and ethics processes [32, 33, 38, 49, 52, 54, 56]; patient recruitment to research [32, 35, 38, 52]; and informatics and information technology [38–40, 46, 47].

Several studies identified complex regulatory processes, such as ethics and research governance, as barriers to translational research, in effect slowing it down [32, 38, 49, 52, 54, 56]. Two surveys of senior researchers working in US medical schools and academic health science centres, found that 38% and 54% of those surveyed identified complex regulatory requirements as particularly challenging for translational research, thus limiting the success of biomedical innovation being translated into benefits for patients [32, 54]. Four qualitative studies, from the field of stem cell research, explain why regulatory processes are particularly challenging for translational research [33, 49, 52, 56]. The rudimentary nature of stem cell research, yet to determine whether stem cell therapy should be defined as a drug or medical technology, complicated regulation in two studies in our sample [33, 49]. Scientists in both these studies reported that this led to confusion over which authority should oversee regulation–a human tissue authority or a food and drug authority. Scientific and technological developments and their accompanying ‘*imagined futures*’ therefore created uncertainty on the part of both scientists and regulators as both parties sought to develop and refine interpretations of rules and regulations, as this extract from a field note illustrates:

[*The representative from the regulator] noted that the views upon this were different across the EU [European Union]*. *He reiterated that it’s hard to know until people make medicinal products what the regulations and guidance should be*. *But the [regulators] are asked to give guidelines anyway even though this is not known*, *and he said ‘it’s circular*, *it goes around and around and around’* [49, p.351].

In another Chinese study, also investigating stem cell research, regulation was complicated by numerous, overlapping regulatory jurisdictions inadvertently promoting inconsistency and minimal conformity with the law, resulting in scientists feeling powerless to change the system [56]. Ethical and social implications of scientific breakthroughs were perceived to add an additional layer of complexity to translational research. Scientists working on stem cells as a potential therapy for leukaemia [33] and diabetes [52] reported making a deliberate effort to follow strict regulatory processes to ensure acceptance and legitimacy of their research [33]. They argued that public attention to the ethical and social implications of stem cell research breakthroughs would limit the move from bench to bedside:

*That then requires a wholesale change of ethical thought as to whether you can put some genetically modified cell back into a human*, *and that’s going to take years and years of legislation* [52, p. 2061]

Basic and clinician scientists articulated mixed views about the role of patient recruitment and collection of patient samples and the implications this has for translational research [32, 35, 38, 52]. In one US study of medical school research leaders, 37% of respondents identified recruitment of research subjects as challenging. When asked whether formal institutional processes and adaptations to counter these challenges had been initiated, 34% identified processes to aid recruitment of research participants, with more than half (54%) reporting that this innovation had a moderate to large effect on the amount of clinical research conducted [32].

Inherent within translational research is the assumption that animal studies will lead to human studies. However, scientists from two studies in our sample [33, 52] commented on a tension between the ‘*relevance of ‘human studies”* and the ‘*rigour of ‘animal experiments”* [52, p.2061]. In one study, based in the UK, basic scientists reported that experiments on animals, due to their availability, were seen as preferable and more likely to lead to outputs in the form of publications. In comparison, experiments on donated human cells were considered to carry a greater risk in terms of output due to difficulties obtaining samples from patients and their families:

*To plan a proper research programme you need regular access to the tissue and you are never going to get that with [human] donor material*, *particularly if the primary objectives are research*, *because the relatives just don’t see it as important to give permission for all this to be used for research*. *Quite rightly I think*, *they don’t feel it’s going to somebody else’s benefit and research is going to be a lower priority*. [52, p.2061]

In the other study, based in China, drugs that had proved effective in animal studies were not effective in human clinical trials because patients’ health conditions were far more complicated than the animal models suggested, requiring scientists to constantly adjust their clinical trial protocols [33].

Solutions proposed to address the problems of recruitment to clinical trials and promote interactions between clinical researchers and potential recruits included community outreach and engagement projects and community advisory boards [38]. Results from one Canadian study suggested that state funded systems of care, such as Canadian Academic Health Science Systems, facilitated translational research due to the availability of a population of patients readily accessible to the researcher [35]. Scientists argued that it is the publicly funded system of care that has made access to patients and their data possible, contrasting this system with that of their neighbours in the United States, whereby the fragmented, privatised systems of care limits the potential for such a valuable resource:

*[The population] is very special and to not take advantage of it would be a huge loss because we can do things here that other people can’t do*. [35, p.721]

Basic and clinician scientists in five studies identified a lack of infrastructure to develop skills in translational research as a further organisational barrier. These included acquiring research skills and access to equipment and effective information technology systems. [38–40, 46, 47]. Effectiveness of clinical and translational science, particularly with regards to accessing information technology systems, was perceived by a number of these scientists to be determined by organisational and leadership factors [46, 47]. For example, a translational research grant awarded to a medical centre was praised by scientists for enabling the widespread provision of infrastructure such as bioinformatics, which was previously unavailable to individual scientists [40].

#### Theme 3: Research versus clinical care

Basic and clinician scientists in four studies identified a perceived cultural divide between basic and clinical science as a key barrier to conducting translational research [44, 52, 54, 55]. In one of these studies, clinician scientists engaged in early stage clinical trials of stem cells understood that such a divide was due to differing scientific worldviews, language and needs among scientists. For example, one clinician scientist said:

*[It’s] still very apparent that we do face*, *this massive void that exists between scientists and clinicians*, *that for most of the part*, *certainly in our area*, *seems to exist*, *with no great understanding of the needs of both*. [55, p.504]

For other scientists, it was no great surprise that basic and clinician scientists were divided given they were educated within different faculties, with differing foundations, management approaches, and hierarchical systems but were then expected to come together to undertake translational research [52, 57]. In contrast, a UK study which interviewed basic and clinician scientists working in cancer genetics found no clear division between clinical practice and research [36]. While, in theory, research and clinical care may be seen as highly differentiated, in practice, as results from this study bear out, the situation may be more complex. The boundary between clinical care and research was characterised as ambiguous, fluid and flexible. This was attributed to the exploratory nature of medical practice sharing similar motivations and procedures to that of clinical research, with both seeking to further knowledge. The fluidity of the relationship was considered to benefit patients (who through research participation gained access to DNA tests not available within clinical services), as well as clinical staff, improving their clinical skills through engagement with research processes [36].

Organisational and structural factors were perceived by clinician scientists in several studies to influence the apparent separation between research and clinical care, which consequently acted as a barrier to translational research [32, 39, 40, 42, 51, 55]. These included: a lack of training in relevant research skills and training being too time intensive [32, 42]; a lack of time to undertake research among clinician scientists due to demanding clinical roles [32, 40, 55]; and the pressure of combining clinical service and research roles [32, 40]. For clinicians working in a community setting, a perceived lower value attributed to research compared to clinical care, with its associated lack of recognition, status and career progression, deterred their participation in research [39]. Clinician scientists reported that having to compete with full-time, non-clinical researchers, perceived as having more time and being better embedded in infrastructures to secure research funding, was a barrier to conducting translational research [42, 51]. This led to the perception among clinician scientists that research was not always considered to be an economically viable activity due to a lack of remuneration for clinical staff’s time and effort to conduct research [39]. The value of conducting translational research for clinician scientists was strongly linked to perceived patient benefit of the research; research considered likely to have low patient benefit was viewed as disruptive to clinical care [36, 39].

In a more positive light, clinician scientists interpreted their role as enabling translational research because they played a key role in bridging the perceived cultural divide between basic and clinical science [33, 39, 41, 44, 52], or by acting as ‘*agents of change*’ due to the strength and breadth of their knowledge [55, p. 503]. Clinician scientists viewed themselves as ‘*boundary-spanners*’ or ‘*important bridges*’ between the laboratory and clinic, acting as mediators and translators in collaboration with ‘*pure*’ clinicians and scientists, whom they relied upon to provide the in-depth clinical and scientific knowledge required for successful translational research [41, p. 542]. In a UK study one clinical-scientist clearly articulated the benefits of adding laboratory work to clinical responsibilities as part of a dual role:

*Mechanistic studies are what I do*. *To do the clinical work [without the lab work] you wouldn’t have learned anything about how it worked and that seemed a bit of a shame to me…you might as well do the study properly*. [44, p.950].

Intellectual differences between basic and clinician scientists in terms of what was considered to constitute ‘legitimate science’ shaped both positive and negative views of translational research among scientists [44, 52, 54]. Basic scientists articulated a greater emphasis on scientific discovery rather than translation research [44, 54], whereas clinician scientists placed more focus on patient-related outcomes to enable translational research [52]. For example, in a US survey of research investigators at a Medical School the most common reason given by basic scientists for not pursuing translational research was because it was not considered central to their research agenda; respondents with PhD degrees were significantly less likely to report they were conducting translational research compared to those with MD or MD/PhD degrees [54].

Basic scientists in five studies tended to hold negative views about translational research as a form of legitimate science [44, 45, 51, 54, 55]. They articulated the view that translational research was not central to their research agenda [45, 54], and perceived clinician scientists as having greater authority to conduct clinical trials to enable translational research practices [55]. Basic scientists perceived reward and career progression as more difficult to achieve in the field of translational research and shared significant concerns about how translational research as a form of science was viewed by their peers and promotion committees [44, 54]. They questioned how they would be able to retain standing in the field if they were publishing in translational research journals, instead of their key disciplinary journals, the former not ‘*sufficiently valued by their peers to form ‘authentic’ knowledge’* [44, p.949]. The downsides of engaging in translational research for non-clinician scientists’ career progression were further emphasised by the tendency for translational research policy to focus on the needs of clinician scientists rather than providing new career structures for basic scientists to work within the requirements of translational research [44, 51]. For example, a basic scientist reported how exasperating she found it that so much attention was paid to ‘*clinician scientists when other professional trajectories might also lead to the establishment of a class of translational investigators’* [51, p.8]. In comparison, most clinician scientists interviewed were more positive about the translational research drive. Translational research was viewed as aligning closer to their own research interests in answering particular health-related questions.

#### Theme 4: Interdisciplinary collaboration

Interdisciplinary collaboration between basic and clinician scientists, but also with other professional groups, was perceived by scientists to facilitate translational research practices [37, 38, 40, 48, 52, 56, 57], by providing opportunities for knowledge exchange [37, 52], offering distinct forms of expertise [48, 56], and creating a working environment which encouraged communication and co-operation between different scientists [38]. Collaboration was seen as being best achieved through multi-disciplinary teams, working throughout the entire research process [40, 52], or through ‘team science’–a field of inquiry to understand and enhance processes which facilitate or inhibit collaboration of researchers across different fields and organisations [37, 48]. For example, one basic scientist working in human embryonic stem cell research in the UK said:

*This is a good place to be because you have got people who make hES cells and this group is a recognised area or centre for expertise in beta cell biology*. *If you put the two together then you progress quite quickly* [52, p.2058].

In contrast, scientists working on stem cell research in China in another study reported that the typical research team structure consisting of ‘*one professor and many students’*, with few, if any, middle ranking researchers was a hindrance to research efficiency and productivity:

*In China, everybody is a professor; everybody works on their own project; there is no connection between groups. Everybody is their own team-leader. Thus it’s hard to make progress [56, p. 197]*.

Perceived facilitators of collaborative working in a translational research network included geographical proximity of different professionals to enable sharing of values and knowledge exchange [34, 43, 52], cohesive bioinformatics and clinical-informatics teams [46] and the appropriate institutional and structural arrangements to accommodate the range of professional expertise required to meet research objectives [56]. Conversely a lack of collaboration between industry and clinical science [50] and poor coordination between translational research and biomedical informatics teams [46] inhibited interdisciplinary collaboration.

The nature of relationships within interdisciplinary collaboration teams was perceived among scientists as another factor shaping collaborative working, which could either enable or hinder translational research. Specifically, translational research was considered to be facilitated by past working relationship practices [43], prior experience of collaboration among scientists across departments [48], and teams with key members who could act as knowledge brokers [43]. Institutions which portrayed themselves as places of collaboration were considered to enable translational research as this collaborative attitude would in turn attract other scientists who believed in collaborative ways of working as an integral part of conducting research [52].

In contrast, scientists identified a number of characteristics that hindered effective collaborative working relationships and practices. Such factors reflected previous professional groupings that encouraged silo-working (where departments or groups do not want to share information or knowledge beyond their group) rather than collaboration in a translational research network [43, 56, 57], and poor leadership or weak mentorship skills of leaders of teams [40, 57]. Other perceived barriers pointed to a lack of experience among scientists collaborating across different departments to form effective teams [48], as well as institutional arrangements which not only inhibit collaboration between research groups within the same institution but also make collaboration between institutions harder [56]. Finally, traditional relationships between academia and industry, with universities as providers of basic research knowledge and industry as translating this knowledge into applications and profits, were viewed by scientists to discourage collaborative working and therefore hinder translational research practices [34, 52].

#### Theme 5: Entrepreneurial science

From a policy perspective, translational research endeavours to improve the health of the nation through the development of effective drugs and treatments, while simultaneously increasing wealth by generating income from a nation’s research capabilities. Translational research therefore comes with health and wealth-based performance measures for those working within translational research environments. A number of papers included in our synthesis suggest that scientists’ research practices are being shaped by this policy agenda [33–35, 41, 44, 51, 53, 57]. They reported a perceived shift in attitudes among scientists and organisations to become ‘entrepreneurial’, thereby facilitating translational research [33–35, 53]. Scientists who considered that both the intellectual and commercial benefits of their research would facilitate translational research, and those with perceived entrepreneurial skills, saw themselves at an advantage to secure funding for translational research.

In addition to the concept of the ‘entrepreneurial scientist’, the concept of the ‘entrepreneurial hospital’ was proposed as a resource for enabling translational research [35]. Scientists in this study considered accessible patient populations, attending publicly funded hospital-university collaborations for their care, as a resource to attract partnerships with industry:

*So the drug company comes in for example to develop a drug with us on a phase I study*. *If we were to extract information at the molecular level for individual patients both of their DNA as well as changes to their tumour*, *we would get a very good understanding as to who’s responding to that treatment*. *We would then be able to advise the drug company as to where we see the best outcomes in terms of patient populations that respond well*. *This*, *presumably would be passed on when it comes to developing their Phase II and Phase III studies and actually improve the likelihood of success* [35, p.721].

Accessible patient populations as a resource had value in terms of rationalising medicine and saving money as well as meeting the commercial aims of translational research. The challenge, as one scientist commented, was ensuring that the mandates of the company and the hospital are both met:

*no matter how they pose the issue of what their mandate is, the company is there to safeguard shareholder value, right? And that usually means sales, how much money have you made? [Our organisation] requires access to those companies’ drugs in many cases to take care of our shareholders, right? But if you look at it as shareholder, what our shareholders are interested in is not making money but being treated appropriately* [35, p.722].

Results from several papers suggest the need for a broader view of translational research whereby not-for-profit institutions have an entrepreneurial role to play [34, 35, 41, 57]. One study identified the need for organisations such as universities to develop the technology to enable translational research opportunities, particularly when industry had lost interest due to patent problems [41].

In two studies, scientists identified the lack of knowledge and interest concerning commercialising research as a barrier to translational research [34, 44]. In a UK study, basic scientists highlighted organisational and structural barriers as limiting their ability to exploit the commercial potential of their research. These were: a lack of awareness of how to patent scientific discoveries or even that this is a necessary step in translational research; and academic funding systems which employ scientists on closed contracts, with institutional measures of performance (and implications for renewal of contracts) based on publication record rather than patents. For example, one basic scientist observed:

*my instinct is I’m wasting my grant time doing that sort of work*, *I’m rewarded for publications not patents* [44, p.949].

However, respondents in a US study highlighted the value of technology transfer offices within a university for taking on industrial collaborations in situations where scientists were less interested in the commercial exploitation of their discoveries [34].

## Discussion

This article reports a systematic review and narrative synthesis of factors that enable or inhibit translational research, from a growing body of empirical studies investigating translational research from the perspective of basic and clinician scientists. To the best of our knowledge this is the only systematic review to have synthesised these factors from the point of view of scientists, a key stakeholder group in translational research. We identified 26 empirical papers from which we synthesised five themes from the perspectives of scientists conducting translational research. These point to areas where policy and practice need development to enhance policy ambitions for translational research to accelerate scientific discoveries into clinical applications with benefits for patients.

The first theme, *concepts of translational research*, identifies a disconnect between linear models of translational research, often adopted at the policy or organisational level, and scientists’ own experience of conducting translational research. Scientists reported that a linear model inhibited key attributes required for successful translation of biomedical innovation, such as interdisciplinary collaboration. Our findings are supported by theoretical work from social scientists who have begun to challenge assumptions underpinning contemporary translational research policy initiatives: the dominant, linear model of translational research does not reflect the reality of how research translation happens in practice [3, 13, 16]. Second, *research processes* at organisational and system levels influenced scientists’ ability to conduct translational research, with complex and lengthy ethical and regulatory research governance processes, difficulties with patient recruitment, and poor access to bioinformatics identified as key barriers limiting translation. Research settings with readily accessible patient populations, for example as part of publicly funded university-hospital collaborations, facilitated patient recruitment for trials and encouraged partnerships with industry, hence enabling translational research. Third, the theme *research versus clinical care* highlighted a cultural divide between science and medicine. Clinician scientists were perceived to bridge this cultural divide, to enable translational research due to their breadth of knowledge, awareness of clinical need and flexible working practices, access to patients for research and ability to run research trials. However, the policy focus on clinician scientists as sole champions for translational research with their perceived ability to bridge the gap between science and medicine was alienating for non-clinician scientists. Fourth, *interdisciplinary collaboration* was thought to enable translational research practices, but depended on the quality of prior and current social relationships. Finally, the theme *entrepreneurial science* indicated that policy drives focusing on health and wealth had encouraged entrepreneurial activity amongst scientists and organisations such as hospitals, although a key challenge was to increase basic scientists’ awareness of the commercial impact of their discoveries.

Social, organisational and structural factors were identified as key contexts across all five themes both enabling and hindering translational research practices. A number of papers in our sample suggest that organisational practices and systems have not kept up with the pace of change in knowledge production brought about by the translational research agenda [42, 44, 51]. For example, the way academic organisations reward work based on individual output from publications and research grants does not match expectations of translational research, which require team working and simultaneously seek to enhance benefits for patients through development of therapies, and to increase the wealth of organisations and nations.

The need to accelerate research as a means to ensure that the endpoints of translational research are met is a view largely articulated in policy, which views the health and medical sciences as an opportunity to increase a nation’s health and wealth. However, basic and clinician scientists in the studies we synthesised did not entirely share this view and warned of unintended consequences resulting from an overly strong focus on translational research and its associated endpoints. They emphasised the need for a broader view of translational research which acknowledges that progress and achievements in science and biomedical innovation may require a longer time frame and that science’s role is concerned with knowledge production and discovery as well as solving problems and producing cures [34, 40, 45, 53].

### Strengths and limitations

A strength of our review is that we used systematic and rigorous methods for searching and synthesising the existing literature on scientists’ perspectives on factors enabling and hindering translation research practices across diverse clinical fields. This included using a number of sources to search the literature and two researchers to select and appraise the literature, and extract and synthesise the data. However, a number of methodological issues could have affected the validity of our findings. The findings of the study are limited by the diversity of terms that are used to describe translational research which may have resulted in a small number of studies being overlooked. Our search strategy focused on English language papers in peer reviewed journals. There are a number of published books concerned with translational research from scientists’ perspectives which were not included due to the design of our search strategy [58]. The omission of this literature, and literature in languages other than in English, may have potentially produced bias or excluded further insights into factors enabling and hindering translational research. We did not exclude studies on the basis of quality. The quality of included studies was mainly high. Seven of the 26 included studies were rated as lower quality (scoring 3 or below). A sensitivity analysis to assess the robustness of our findings, suggests that removing the seven lower quality studies does not alter the thematic categories we derived from the synthesis.

### Implications for Policy and Practice

Some of the barriers we have identified in our review have been reported in opinion pieces, (for example inadequate infrastructure, the need for a research culture that facilitates collaboration [2,5]). This study investigated translational research practices across health domains, scientific disciplines, and countries, and included the perspectives of wide range of scientists. Our findings substantiate and add to known concerns, highlighting that new strategies are required to maximise the potential for translational research to deliver benefits to patients. The findings of our review point to a number of policy implications for those seeking to better realise the potential for translational research.

First, our findings question the usefulness of the pipeline, linear model of translational research. This does not reflect how scientists conducting translational research describe what they do. We call for policy and institutions promoting translational research to refine their conceptual models of translational research to one that is more reflective of scientists’ experiences of translational research, fostering greater buy in from all types of scientists. Our review provides evidence that scientists’ consider collaboration as a key ingredient for successful translational research and that viewing translational research as a circular or iterative process encourages collaboration. Therefore a refined model of translational research may foster greater reciprocal interaction between the laboratory and the clinic, in turn attracting further collaborations.

Second, the challenge of undertaking translational research requires change at organisational and institutional levels. This will necessitate streamlining regulatory and governance processes and facilitating access to infrastructures such as bioinformatics, in order to reduce delays to translational research. The conflicting needs of academic and hospital institutions, whose primary goals are respectively to foster academic achievement and to provide healthcare solutions, will need to be resolved. Stronger leadership and integrated institutions are required with single managerial, governance and administrative structures that afford appropriate weight to these conflicting needs in order to prioritise translational research.

Third, the end points of translational research are frequently discussed as contributing to a nation’s health and wealth, although it is not always clear from policy statements which component is being prioritised and, as stated earlier, whether these aspirations have been met through the push for translational research. A broader policy focus on translational research would facilitate the contribution to biomedical innovation from public sector institutions as well as industry to drive translational research, particularly in situations when industry cannot participate due to problems with patents or when public sector institutions can establish the market before it is viable for the commercial sector to enter. In line with other literature on the potential for translational research, findings from our synthesis suggest that translational research provides opportunities for realigning relations between citizens, patients, healthcare providers, pharmaceutical companies and biomedical researchers [16].

## Supporting Information

S1 PRISMA Checklist Preferred Reporting Items for Systematic Reviews and Meta-Analyses checklist. (DOC)
